# Oxidized LDL Increase the Proinflammatory Profile of Human Visceral Adipocytes Produced by Hypoxia

**DOI:** 10.3390/biomedicines9111715

**Published:** 2021-11-18

**Authors:** Concepción Santiago-Fernández, Flores Martín-Reyes, Monica Tome, Carolina Gutierrez-Repiso, Diego Fernandez-Garcia, Luis Ocaña-Wilhelmi, Jose Rivas-Becerra, Franz Tatzber, Edith Pursch, Francisco J. Tinahones, Eduardo García-Fuentes, Lourdes Garrido-Sánchez

**Affiliations:** 1 Unidad de Gestión Clínica de Aparato Digestivo, Instituto de Investigación Biomédica de Málaga (IBIMA)/Universidad de Málaga, Hospital Universitario Virgen de la Victoria, 29010 Málaga, Spain; conchisantiagofernandez@gmail.com (C.S.-F.); floresmarey@hotmail.com (F.M.-R.); 2 Unidad de Gestión Clínica de Endocrinología y Nutrición, Hospital Regional Universitario, 29010 Málaga, Spain; doctoratome@gmail.com; 3 Unidad de Gestión Clínica de Endocrinología y Nutrición, Instituto de Investigación Biomédica de Málaga (IBIMA)/Universidad de Málaga, Hospital Universitario Virgen de la Victoria, 29010 Málaga, Spain; carogure@hotmail.com (C.G.-R.); diegofernandezgarcia@hotmail.com (D.F.-G.); lourgarrido@gmail.com (L.G.-S.); 4 CIBER Fisiopatología Obesidad y Nutrición (CIBERobn), Instituto Salud Carlos III, 29010 Málaga, Spain; 5 Unidad de Gestión Clínica de Cirugía General y Digestiva, Hospital Universitario Virgen de la Victoria, 29010 Málaga, Spain; luisowilhelmi@hotmail.com; 6 Unidad de Gestión Clínica de Cirugía General, Digestiva y Trasplantes, Hospital Regional Universitario, 29010 Málaga, Spain; doctopep@hotmail.com; 7 Otto Loewi Research Center, Division of Immunology and Pathophysiology, Medical University of Graz, 8010 Graz, Austria; franz@tatzber.at; 8 Institute of Biochemical Engineering, University of Applied Sciences Technikum-Wien, 1200 Vienna, Austria; werner.pursch@aon.at; 9 CIBER Enfermedades Hepáticas y Digestivas (CIBERehd), Instituto Salud Carlos III, 29010 Málaga, Spain

**Keywords:** hypoxia, scavenger receptors, oxidized LDL, adipocytes, morbid obesity

## Abstract

Background: Little is known about the effects of hypoxia on scavenger receptors (SRs) levels in adipocytes. We analyzed the effect of morbid obesity and hypoxia on SRs and inflammation markers in human visceral adipocytes and whether ox-LDL modify the inflammatory profile produced by hypoxia. Methods: We studied in 17 non-obese and 20 subjects with morbid obesity (MO) the mRNA expression of HIF-1α, SRs (LOX-1, MSR1, CL-P1 and CXCL16), IL6 and TNFα in visceral adipocytes and the effect of hypoxia with or without ox-LDL on visceral in vitro-differentiated adipocytes (VDA). Results: HIF-1α, TNFα, IL6, LOX-1, MSR1 and CXCL16 expression in adipocytes was increased in MO when compared with those in non-obese subjects (*p* < 0.05). The expression of most of the inflammatory markers and SRs gene correlated with HIF-1α. In VDA, hypoxia increased TNFα, IL6, MSR1, CXCL16 and CL-P1 (*p* < 0.05) in non-obese subjects, and TNFα, IL6, MSR1 and CXCL16 (*p* < 0.05) in MO. Silencing HIF-1α prevented the increase of TNFα, IL6, LOX-1, MSR1, CL-P1 and CXCL16 expression (*p* < 0.05). The combination of hypoxia and ox-LDL produced higher TNFα expression (*p* = 0.041). Conclusions: Morbid obesity and hypoxia increased SRs and inflammatory markers in visceral adipocytes. In a hypoxic state, ox-LDL increased the proinflammatory response of visceral adipocytes to hypoxia.

## 1. Introduction

Obesity is associated with an increase of adipose tissue hypoxia [1,2]. Previously, a study showed that the visceral adipose tissue (VAT) oxygenation level is negatively associated with obesity indicators such as waist circumference, waist-to-hip ratio and body mass index (BMI) [3]. Hypoxia mainly mediates its effects through the activation of hypoxia-inducible factor 1-α (HIF-1α) [4]. Current studies link hypoxia to adipose tissue inflammation [5] and an alteration of adipocytokines synthesis, which are involved in the regulation of angiogenesis, cell proliferation, apoptosis, inflammation and insulin resistance [4,6,7].

Hypoxia is an important factor for transcriptional regulation of cell metabolism. HIF-1α modulates the function of phagocytic cells, mainly macrophages, by stimulating surface receptors such as scavenger receptors (SRs) for oxidized low-density lipoproteins (ox-LDL) [8]. A previous study showed that hypoxia reoxygenation of cardiomyocytes induces lectin-like oxidized low-density lipoprotein receptor-1 (LOX-1), a type of SR [9]. However, there are different SRs for ox-LDL [10] which are associated with most of the proatherogenic risk factors, including obesity, type 2 diabetes, dyslipidemia and metabolic syndrome [11]. The ox-LDL is mainly removed from circulation by different SRs such as CD36, macrophage scavenger receptor (MSR1 or SR-AI), LOX-1, SR for phosphatidyl serine and ox-LDL (SR-PSOX, also called C-X-C motif chemokine ligand 16 (CXCL16)) and the collectin subfamily member 12 (CL-P1). These SRs are responsible for approximately 90% of the uptake of ox-LDL [12]. Moreover, it is known that the effects of this type of lipoproteins on the regulation of cell metabolism are mediated by SRs [13]. Thus, the study of these SRs is of great importance because ox-LDL can modulate inflammatory responses and atherosclerosis [14].

Most of these SRs have been identified in macrophages and endothelial cells, but their presence in adipocytes is not very well-known [15]. SR-A and LOX-1 mRNA levels are significantly higher in the stromal vascular fraction compared to those in the adipocyte fraction from subcutaneous adipose tissue (SAT), whereas CD36 mRNA is more highly expressed in adipocytes [16]. However, the studies on the effect of hypoxia on LOX-1 expression or other SRs in human adipocytes are very scarce [16]. If the expression of SRs in visceral adipocytes were altered by hypoxia, this could promote a proatherogenic and proinflammatory profile of this type of cells. This is of special importance in those diseases in which ox-LDL is a pathological factor, such as cardiovascular diseases, type 2 diabetes mellitus, obesity and other metabolic diseases [17].

It is known that ox-LDL is uptaken by visceral in vitro-differentiated adipocytes, and its increase stimulates the proinflammatory profile of these visceral adipocytes [18]. These effects are mediated by the binding of ox-LDL to its receptors, the SRs. This increase in the formation of proinflammatory cytokines by adipocytes plays an important role in the development of cardiometabolic and cardiovascular diseases [19]. In this context, hypoxia could also stimulate proatherosclerotic processes and proinflammatory signaling. However, the molecular mechanism of this relation in adipocytes remains unclear [20]. This type of cells is closely involved in the regulation of insulin resistance, type 2 diabetes mellitus, chronic low-grade inflammation and atherosclerosis [21].

The aims of this study were to analyze in human visceral adipocytes (a) whether morbid obesity modified SRs expression, (b) whether hypoxia could produce an increase of SRs expression, as we had previously demonstrated for ox-LDL [18], (c) the effect of hypoxia on inflammation markers and (d) whether ox-LDL could increase the inflammatory response produced by hypoxia.

## 2. Materials and Methods

### 2.1. Subjects

We evaluated 20 subjects with morbid obesity (BMI 56.1 ± 8.6 kg/m^2^) who underwent biliopancreatic diversion of Scopinaro (BPD) at the Virgen de la Victoria University Hospital, Malaga (Spain) [22,23] and 17 non-obese subjects (BMI 22.5 ± 1.7 kg/m^2^) who were chosen from those that underwent a scheduled laparoscopic cholecystectomy at the Regional University Hospital, Malaga (Spain) (Table 1). No subjects underwent surgery for the sole purpose of obtaining samples for this study. Subjects were excluded if they were receiving insulin or hypoglycemic agents, had cardiovascular disease, arthritis, acute inflammatory disease or infectious disease. Samples were processed and frozen immediately after their reception in the Virgen de la Victoria University Hospital Biobank (Andalusian Public Health System Biobank) (Spain). The objectives of the study were explained to all participants, non-obese subjects and those with morbid obesity. They gave their written informed consent. The study was carried out in accordance with the Code of Ethics of the World Medical Association (Declaration of Helsinki) and was approved by the Malaga Provincial Research Ethics Committee, Spain (CEI_CP13-00188).

### 2.2. Laboratory Measurements

Blood samples from all subjects were collected after a 10-h fast. The serum was separated and immediately frozen at −80 °C. Serum biochemical variables were measured in duplicate as previously described [23,24]. HOMA-IR was also calculated.

### 2.3. Mature Adipocyte Isolation

VAT was obtained and washed in physiological saline. VAT adipocytes were isolated after a digestion of VAT as previously described, immediately frozen in liquid nitrogen and maintained at −80 °C until analysis [22,23,25,26].

### 2.4. In Vitro-Differentiated Visceral Adipocyte Culture

The pellet of the digested human VAT (stromal vascular fraction, SVF) from non-obese subjects (*n* = 5) and morbidly obese subjects (*n* = 5) was washed twice with DMEM (Merck KGaA, Darmstadt, Germany), treated with eBioscence™ Red Blood Cell Lysis Buffer (Invitrogen, Waltham, MA, USA) and used for the differentiation of human mesenchymal stem cells (HMSC) into adipocytes as previously described [18]. At day 15 of differentiation, the culture medium on which adipocytes were differentiated was changed [18] and adipocytes were incubated for 24 h at 37 °C in 95% air and 5% CO_2_ (normoxic conditions) or placed in a hypoxic chamber (Billups-Rothenberg, Dell Mar, CA, USA) at 37 °C and 1% O_2_, 5% CO_2_ and 94% N_2_ (hypoxic conditions) [1]. After incubation, the adipocytes were harvested and frozen at −80 °C until analysis. Each treatment was performed in triplicate.

In a second experiment, the SVF of VAT from non-obese subjects (*n* = 5) was cultured as previously described until day 15 of differentiation in normoxia conditions [27]. At day 15, the culture medium was replaced with a fresh medium. The transfection was made with a siRNA against HIF-1α (s6539) (NM_001243084.1, NM_001530.3, NM_181054.2) (Thermo Fisher Scientific Inc, Waltham, MA, USA) and DharmaFECT^TM^ Transfection Reagent (GE Healthcare, Buckinghamshire, UK), according to the manufacturer’s instructions, with experimental control samples transfected with Silencer^®^ Select Negative Control siRNAs (Thermo Fisher Scientific Inc, Waltham, MA, USA). The cells were incubated for 24 h at 37 °C in 95% air and 5% CO_2_ (normoxic conditions) [18]. After these 24 h, the cells were incubated for another 24 h at 37 °C in 95% air and 5% CO_2_ (normoxic conditions) or placed in a hypoxic chamber at 37 °C and 1% O_2_, 5% CO_2_ and 94% N_2_ (hypoxic conditions). Afterwards, the cells were harvested and frozen at −80 °C until analysis. Each treatment was performed in triplicate.

In a third experiment, the SVF of VAT from non-obese subjects (*n* = 5) was cultured as previously described until day 15 of differentiation in normoxia conditions [27]. On day 15, the culture medium was changed, and adipocytes were incubated for 24 h at 37 °C in 95% air and 5% CO_2_ (normoxic conditions) or placed in a hypoxic chamber at 37 °C and 1% O_2_, 5% CO_2_ and 94% N_2_ (hypoxic conditions). After these 24 h of incubation, 0 and 50 µg/mL of malondialdehyde-modified human LDL (ox-LDL) (0 and 50 ug protein/mL of malondialdehyde-modified ox-LDL) (MyBioSource, Inc., San Diego, CA, USA) were added [28] to the normoxia and hypoxia conditions. After another 24 h of incubation, the adipocytes were harvested and frozen at −80 °C until analysis. Each treatment was performed in triplicate.

### 2.5. RNA Extraction and Real-Time Quantitative PCR

Total RNA from frozen human mature adipocytes and in vitro-differentiated adipocytes were isolated using an RNeasy Lipid Tissue Mini Kit (Qiagen, GmbH, Hilden, Germany) as previously described [23,27,29]. Gene expression was assessed by real-time PCR using an Applied Biosystems 7500 Fast Real-Time polymerase chain reaction System (Applied Biosystems, Darmstadt, Germany). The reactions were carried out in duplicate for all genes using specific TaqMan^®^ Gene Expression Assays: MSR1 (Hs00234007_m1, RefSeq. NM_002445.3, NM_138715.2, NM_138716.2), CXCL16 (Hs00222859_m1, RefSeq. NM_001100812.1, NM_022059.2), LOX-1 (Hs01552593_m1, RefSeq. NM_001172632.1, NM_001172633.1, NM_002543.3), CL-P1 (Hs00560477_m1, RefSeq. NM_130386.2), HIF-1α (Hs00153153_m1, RefSeq. NM_001243084.1, NM_001530.3, NM_181054.2), GLUT1 (Hs00892681_m1, RefSeq. NM_006516.2), IL6 (Hs00174131_m1; RefSeq: NM_000600.3) and TNFα (Hs00174128_m1; RefSeq: NM_000594.3). The threshold cycle (Ct) value for each sample was normalized with the expression of cyclophilin A (*PPIA*) (4326316 E, RefSeq. NM_021130.3) [1]. SDS software 2.3 and RQ Manager 1.2 (Applied Biosystems, Foster City, CA, USA) were used to analyze the results with the comparative Ct method (2^−^^ΔCt^).

### 2.6. Statistical Analysis

The statistical analysis was done with SPSS (Version 11.5 for Windows; SPSS, Chicago, IL, USA). Differences between the two groups were compared by the Mann–Whitney test. Differences between two related variables were analyzed by the Wilcoxon test. Differences between conditions of ox-LDL/hypoxia incubations were made with a repeated-measure ANOVA. Spearman correlation coefficients were calculated to estimate the linear correlations between variables. Values were considered to be statistically significant when *p* ≤ 0.05. The results are given as the mean ± SD.

## 3. Results

### 3.1. HIF-1α and Inflammation Markers Are Increased in Visceral Adipocytes from MO

**Figure 1:**
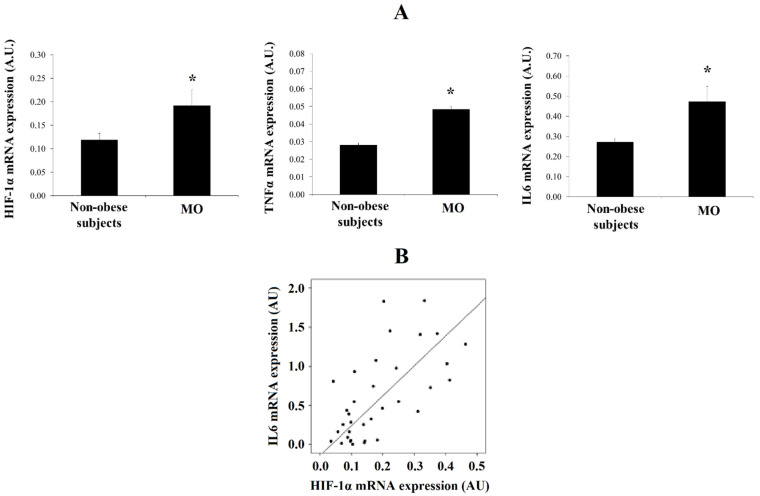
(**A**) Levels of mRNA expression of HIF-1α, TNFα and IL6 in human visceral adipocytes from non-obese subjects and patients with morbid obesity (MO). Data are the mean ± SEM. * *p* < 0.05: significant differences between non-obese subjects and MO. (**B**) Significant correlation found in visceral mature adipocytes between the mRNA expression of HIF-1α and IL6.

HIF-1α (*p* = 0.013), IL6 (*p* = 0.043) and TNFα (*p* = 0.049) mRNA expression in mature adipocytes was increased in MO when compared with that in non-obese subjects (Figure 1A). HIF-1α expression only significantly correlated with IL6 expression (r = 0.652, *p* = 0.002) (Figure 1B).

### 3.2. SRs Levels Are Increased in Visceral Adipocytes from MO

**Figure 2:**
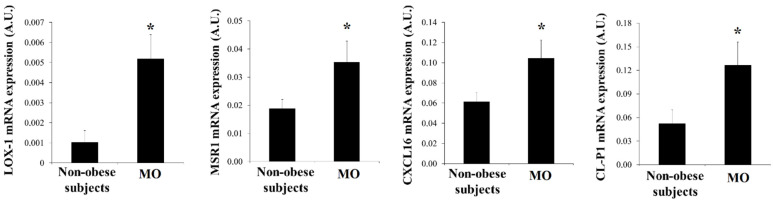
Levels of mRNA expression of scavenger receptors in human visceral adipocytes from non-obese subjects and patients with morbid obesity (MO). Data are the mean ± SEM. * *p* < 0.05: significant differences between non-obese subjects and MO.

Since there are few data on the presence of SRs in human visceral adipocytes, we wanted to analyze the expression of these SRs. LOX-1 (*p* = 0.008), MSR1 (*p* = 0.041), CXCL16 (*p* = 0.027) and CL-P1 (*p* = 0.037) mRNA expression was increased in mature adipocytes from MO when compared with that in non-obese subjects (Figure 2).

### 3.3. SRs Levels Are Associated with Inflammation Markers

**Figure 3:**
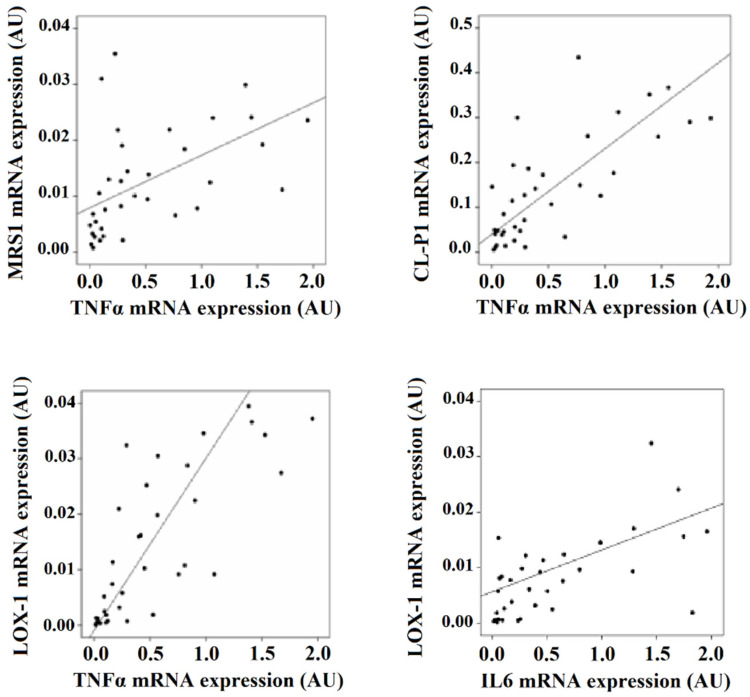
Significant correlations found in visceral mature adipocytes between the mRNA expression of scavenger receptors with HIF-1α and IL6.

In visceral adipocytes, IL6 expression correlated with LOX-1 expression (r = 0.670, *p* = 0.002). TNFα correlated with LOX-1 (r = 0.761, *p* = 0.001), MSR1 (r = 0.580, *p* = 0.005) and CL-P1 expression (r = 0.501, *p* = 0.021) (Figure 3).

### 3.4. SRs Levels Are Associated with HIF-1α

**Figure 4:**
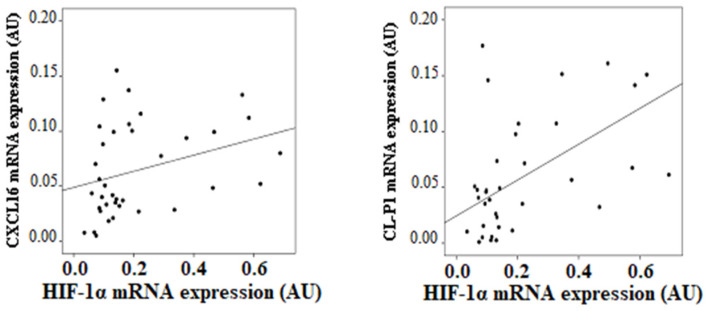
Significant correlations found in visceral mature adipocytes between the mRNA expression of HIF-1α and scavenger receptors.

In visceral adipocytes, HIF-1α expression correlated with CL-P1 (r = 0.470, *p* = 0.018) and CXCL16 expression (r = 0.403, *p* = 0.034), both after adjusting by BMI (Figure 4). No other significant associations were found in mature adipocytes (data not shown).

### 3.5. Hypoxia Modifies SRs Expression of In Vitro-Differentiated Visceral Adipocytes

**Figure 5:**
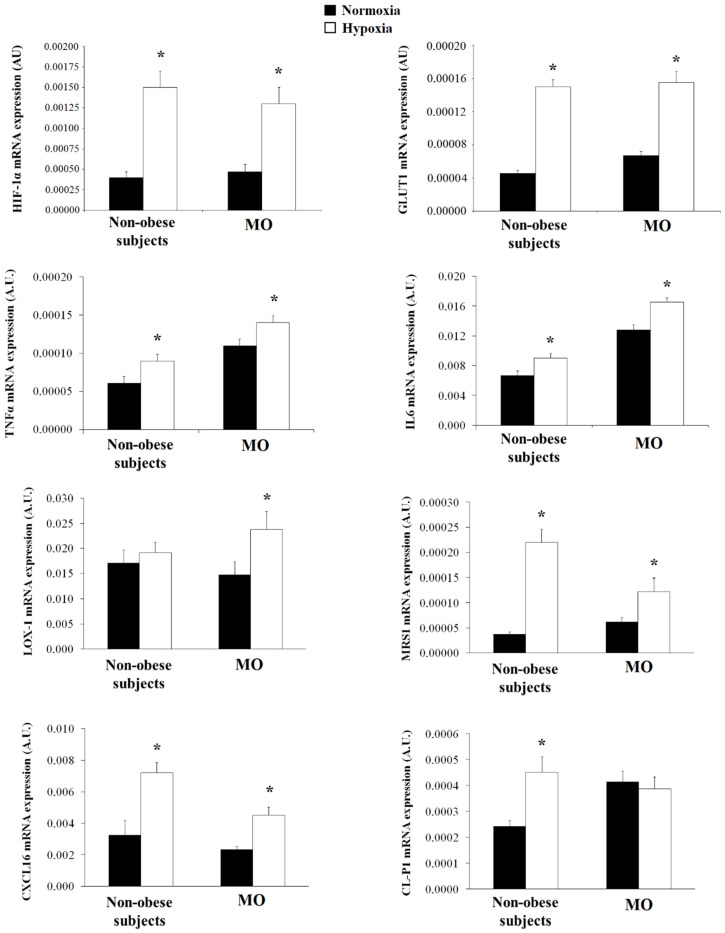
Levels of HIF-1α, GLUT1, IL6, TNFα and scavenger receptors mRNA expression of in vitro-differentiated adipocytes obtained from human mesenchymal stem cells of visceral adipose tissue (VAT) from non-obese subjects (*n* = 5) and patients with morbid obesity (*n* = 5) (MO) incubated in normoxia (■) and hypoxia (□) conditions for 24 h. Each treatment was performed in triplicate. Data are the mean ± SEM. * *p* < 0.05: significant differences between the normoxia and hypoxia conditions.

Since HIF-1α was associated with the SRs level, we want to analyze whether hypoxia was involved in the regulation of SRs expression in visceral adipocytes. First, hypoxia produced a significant increase of HIF-1α and GLUT1, hypoxia markers, in non-obese (*p* = 0.017 and *p* = 0.003, respectively) and in MO (*p* = 0.015 and *p* = 0.002, respectively) (Figure 5). Hypoxia produced a significant increase of IL6 (*p* = 0.043), TNFα (*p* = 0.046), MSR1 (*p* = 0.002), CXCL16 (*p* = 0.026) and CL-P1 expression (*p* = 0.009) in non-obese subjects (Figure 5), and IL6 (*p* = 0.047), TNFα (*p* = 0.049), MSR1 (*p* = 0.041), CXCL16 (*p* = 0.035) and LOX-1 (*p* = 0.025) in MO (Figure 5).

### 3.6. Silencing HIF-1α Counteracts the Effects of Hypoxia on SRs Expression Levels

**Figure 6:**
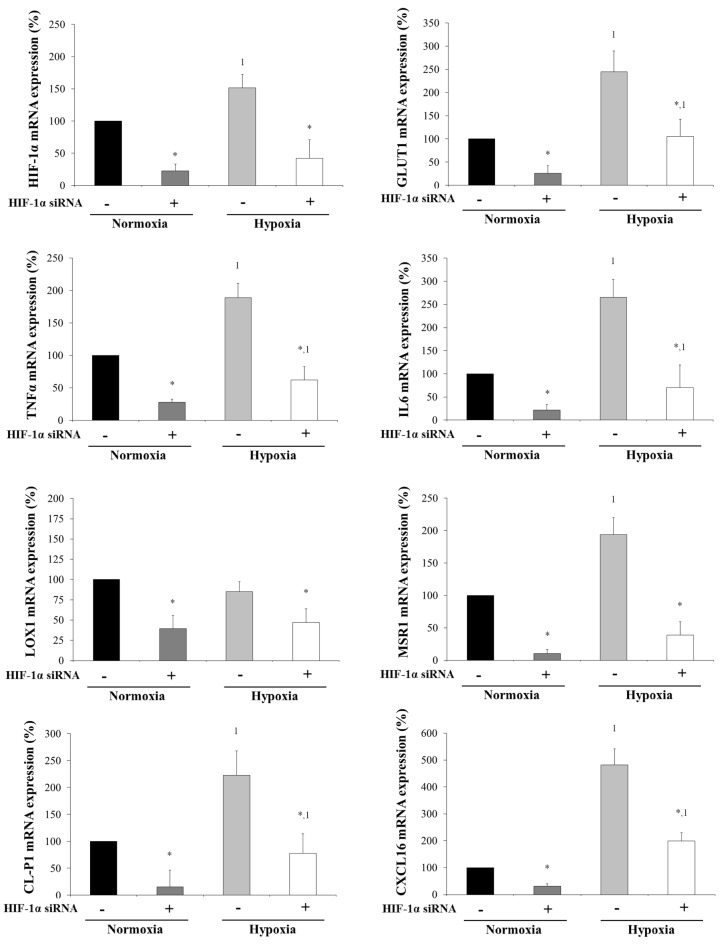
Levels of HIF-1α, GLUT1, TNFα, IL6 and scavenger receptors mRNA expression (%) of in vitro-differentiated adipocytes obtained from human mesenchymal stem cells of visceral adipose tissue (VAT) from non-obese subjects (*n* = 5) incubated in normoxia and hypoxia conditions when HIF-1α was silenced as described in the Materials and Methods section. Briefly, at day 15 of adipocyte differentiation, the transfection was made with a siRNA (s6539) and DharmaFECT^TM^ Transfection Reagent. After 24 h in the normoxia condition, the cells were incubated for another 24 h in normoxia or hypoxia conditions. Cells were harvested and frozen at −80 °C until analysis. Each treatment was performed in triplicate. Data are the mean ± SEM. Results are shown as a percentage with regard to normoxia condition (100%) without hypoxia and HIF-1α silencing. * *p* < 0.05: significant differences between silenced and not silenced HIF-1α conditions. ^1^ *p* < 0.05: significant differences between normoxia and hypoxia conditions when HIF-1α is or is not silenced.

Since hypoxia modified SRs expression levels, we wanted to know whether HIF-1α silencing had an effect on SRs levels. Since the behavior of hypoxia markers (HIF-1α and GLUT1) was similar in hypoxia in the incubations of visceral adipocytes from non-obese and MO (Figure 5), we silenced HIF-1α only in those in vitro-differentiated adipocytes from non-obese subjects. First, we checked the validity of the silencing method, finding that HIF-1α silencing produced a significant decrease of its expression in both normoxia and hypoxia conditions (Figure 6). Subsequently, our results showed that HIF-1α silencing produced a significant decrease of GLUT1, another hypoxia marker (*p* < 0.05), inflammation markers (TNFα and IL6) (*p* < 0.05) and SRs (LOX1, MSR1, CXCL16 and CL-P1) (*p* < 0.05) (Figure 6) in the normoxia and hypoxia conditions.

### 3.7. Combined Effects of Hypoxia and Ox-LDL on Inflammation Marker Levels of In Vitro-Differentiated Visceral Adipocytes

**Figure 7:**
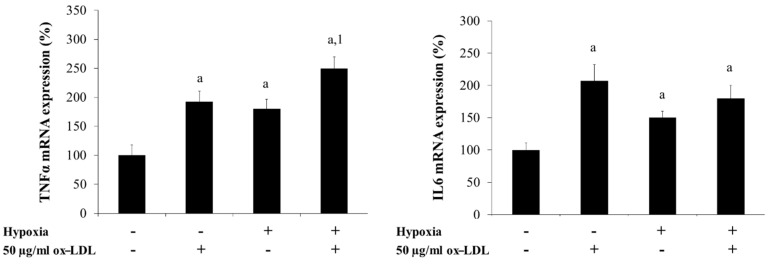
Levels of mRNA expression (%) of TNFα and IL6 of in vitro-differentiated adipocytes obtained from human mesenchymal stem cells of VAT from non-obese subjects (*n* = 5) incubated in hypoxia conditions with ox-LDL. Briefly, at day 15 of adipocyte differentiation, the cells were incubated for 48 h in normoxia or hypoxia conditions. After the first 24 h of incubation, 0 and 50 µg/mL of human ox-LDL were added. After another 24 h of incubation, the adipocytes were harvested and frozen at −80 °C until analysis. Each treatment was performed in triplicate. Data are the mean ± SEM. . Results are shown as a percentage with regard to normoxia condition (100%) without hypoxia and ox-LDL. ^a^ *p* < 0.05: significant differences respect to the normoxia condition without 50 µg/mL ox-LDL. ^1^ *p* < 0.05: significant differences between hypoxia and hypoxia + ox-LDL conditions.

Since hypoxia produced an increase of SRs in in vitro-differentiated adipocytes and SRs correlated with inflammation markers, we wanted to know whether ox-LDL, due to the increase of SRs, could increase the inflammatory response produced by hypoxia. We performed incubations of in vitro-differentiated adipocytes from HMSC of VAT from non-obese subjects in hypoxia conditions (48 h), with or without 50 µg/mL ox-LDL for 24 h as described in the Materials and Methods section. As expected, hypoxia produced an increase of IL6 (*p* = 0.038) and TNFα expression (*p* = 0.012) (Figure 7). The presence of hypoxia and 50 µg/mL ox-LDL for 24 h produced a significant increase of TNFα (*p* = 0.003) and IL6 expression (*p* = 0.045) in normoxia conditions, and a significant increase of TNFα expression in hypoxia conditions (*p* = 0.041) (Figure 7).

## 4. Discussion

In this study, we found that visceral mature adipocytes from patients with morbid obesity had increased expression of HIF-1α, inflammation markers and different SRs. Most of the SRs significantly correlated with HIF-1α, suggesting an association with the hypoxia state. This association is partially confirmed by the in vitro incubation in hypoxia, resulting in an increase of most SRs, although it depends on the type of receptor and the type of patients from which the differentiated visceral adipocytes come. When we silenced HIF-1α, the expression of SRs decreased. Moreover, the combination of hypoxia and ox-LDL produced higher TNFα expression. These results suggest that SRs seem to be involved in the inflammatory response of visceral adipocytes to hypoxia, which is increased by ox-LDL.

In a previous study, we found that patients with morbid obesity had high VAT HIF-1α expression [1]. However, to date, most of these studies on HIF-1α have been performed in total adipose tissue or in macrophages. Our results also showed this situation in mature adipocytes, where there was an increase of HIF-1α and GLUT1. These findings could be related to the intermittent hypoxia to which this type of patients is usually subjected. The close relationship between obesity and obstructive sleep apnea (OSA) is known [30], in which an upregulation of serum HIF-1α protein was found [31] with a decrease after two months of continuous positive airway pressure treatment [32]. However, we have no data on sleep apnea syndrome in our patients. We also found that hypoxia seems to be associated with an increase of the inflammatory profile of visceral adipocytes. HIF-1α was positively associated with IL6. This inflammation marker and TNFα were also increased in patients with morbid obesity. This association between hypoxia and inflammation was reinforced by the increase of those inflammation markers in visceral adipocytes when they were subjected to hypoxia and its decrease after HIF-1α silencing. This agrees with previous studies showing an upregulation of inflammatory genes in hypoxia [33,34,35,36], although in our case, macrophages were not responsible. We suggest that visceral adipocytes from patients with morbid obesity are in hypoxia and that these cells could participate in the upregulation of the inflammation found in the hypoxia state.

On the other hand, we also found the presence of SRs expression in visceral adipocytes, higher in patients with morbid obesity than in non-obese subjects. There are few studies showing the mRNA expression of different SRs in human adipocytes [15,16,37,38]. In addition, we found a positive correlation between HIF-1α and CL-P1 and CXCL16, which suggests an association between hypoxia and SRs. The absence of a significant association with other SRs might be due to different confounding factors, as we discuss later. However, we demonstrated in vitro that hypoxia induced upregulation of SRs in non-obese subjects and in patients with morbid obesity. Moreover, the silencing of HIF-1α produced a decrease of these SRs. These findings show that visceral adipocytes express SRs and that hypoxia could be a factor involved in the regulation of SRs in this type of cells.

CD36 is the most-studied SR, even in adipocytes, where it is the main SR [16]. It is also involved in other functions as a facilitator of long chain fatty acid uptake [38,39]. However, studies analyzing other SRs in adipocytes and how they can be modified in certain pathophysiological conditions are much scarcer. Studies involving these receptors may contribute to a more complete understanding of the effects of ox-LDL on adipocytes. Among these other SRs, LOX-1 may be one of the preferential receptors through which ox-LDL is internalized, as studies in other cell types seem to suggest [40]. LOX-1 also plays an important role in proinflammatory signaling in other types of cells [41,42,43]. Our findings show an increase of LOX-1 expression in visceral adipocytes from patients with morbid obesity, who also have an increase of HIF-1α, as Crucet et al. found in hypoxic macrophages, thereby enhancing ox-LDL uptake [43]. Moreover, LOX-1 is also found in other cell types to be overexpressed in endoplasmic reticulum stress conditions, as when human aortic smooth muscle cells were incubated with ox-LDL. In this sense, morbid obesity is a pathology in which there is an increase of oxidative stress [44]. However, in vitro-differentiated adipocytes did not upregulate LOX-1 mRNA under hypoxia. These differences between the in vivo and in vitro experiment could be due to the fact that in vivo, LOX-1 expression could be induced by several proinflammatory and proatherogenic stimuli, and by oxidative stress, not by hypoxia [45].

Regarding MSR1, we found a higher expression in patients with morbid obesity, an increase in hypoxia and a positive correlation with HIF-1α. However, other studies have shown a decrease of MSR1 in hypoxic macrophages [46,47]. This suggests that the regulation of MSR1 expression could be different depending on the type of cells. Other unknown factors different to hypoxia could be involved in the increased MSR1 expression found in visceral adipocytes, such as increased endoplasmic reticulum stress [48].

The relation of hypoxia with other SRs, such as CXCL16 and CL-P1, has not been studied in adipocytes. We show for the first time that these two SRs are higher in patients with morbid obesity and in hypoxia. Although the regulation of CXCL16 and CL-P1 expression has not yet been clarified, HIF-1α could be involved as our in vitro results show. However, CL-P1 expression could be also increased by oxidative stress provoked by hypoxia in human adipocytes [49].

Our findings also show that there were no significant differences in the in vitro expression of these genes in differentiated adipocytes in normoxia conditions between those derived from non-obese patients and from those with morbid obesity, unlike what happens in vivo. This could be because the in vitro culture conditions were the same for the samples from both types of subjects. However, the environment surrounding such samples in vivo is different, i.e., the state of low-grade chronic inflammation associated with obesity or different hormone levels.

All these SRs are mediators of the effects of ox-LDL on the regulation of cell metabolism [13]. Different studies have shown a significant association between ox-LDL and circulating levels of proinflammatory cytokines [50,51], with the underlying mechanism not fully clarified. In this line, another point that we wanted to analyze in this study was to verify the possible existence of a synergy between the effect of hypoxia and high levels of ox-LDL on the inflammatory profile of visceral adipocytes. Previously, we showed an increased level of serum ox-LDL in morbid obesity [24] and that ox-LDL could increase LOX-1 expression and sensitize adipocytes to a more proinflammatory phenotype [18]. In the current study, we have found that the combination of hypoxia and ox-LDL produced higher TNFα and IL6 expression, although this increase was only significant with TNFα. This could have important repercussions on the regulation of the inflammatory state present in patients with morbid obesity.

One of the limitations of our study is the lack of information on protein levels, since the expression of SRs was low in adipocytes, and samples were collected exclusively for mRNA expression analysis. However, we have demonstrated for the first time that these SRs were expressed in visceral mature adipocytes, regulated by in vitro hypoxia. Additionally, it would be interesting to analyze the presence of SRs in macrophages, although this study has been focused on analyzing its presence in adipocytes, which was not studied to date. Moreover, it would have been interesting to have data on the presence of sleep apnea syndrome in the patients included in this study. To further clarify the role of ox-LDL and hypoxia on SRs and adipocyte metabolism, an experiment with HIF-1α silenced and ox-LDL would be necessary to unravel if hypoxia and ox-LDL share the same activation pathway for the overexpression of SRs.

## 5. Conclusions

Together, our results show that visceral adipocytes from patients with morbid obesity seem to be in a hypoxia state, which could be a factor involved in the regulation of SRs and inflammation in this type of cells. Hypoxia resulted in a significant increase of most of the SRs, which were downregulated when HIF-1α is silenced. Despite the abundant literature on the role of SRs in foam cell formation, macrophages and in atherosclerotic lesions, little is known about its involvement in the regulation of the inflammatory state of visceral adipocytes. In this hypoxia state, high levels of ox-LDL increase the inflammatory response of visceral adipocytes. Together, the increase of SRs could cause adipocytes to behave as an inflammatory source and could contribute to the low-grade inflammation present in obesity. Further studies are necessary to verify the role that each SR may have in the metabolism of visceral adipocytes.

## Figures and Tables

**Table 1:** Anthropometric and biochemical variables in the non-obese and morbidly obese subjects.

	Non-Obese Subjects (*n* = 21)	Patients with Morbid Obesity (*n* = 26)
**Sex (male/female)**	9/12	9/17
**Age (years)**	44.6 ± 16.1	38.9 ± 10.5
**Weight (Kg)**	63.2 ± 9.5	148.2 ± 28.3 ^2^
**BMI (kg/m^2^)**	22.5 ± 1.7	56.1 ± 8.6 ^2^
**Waist (cm)**	82.1 ± 9.9	141.9 ± 17.4 ^2^
**Hip (cm)**	96.5 ± 5.4	155.6 ± 14.8
**Glucose (mg/dL)**	89.4 ± 14.0	94.1 ± 11.7
**Cholesterol (mg/dL)**	202.8 ± 34.4	195.2 ± 41.7
**Triglycerides (mg/dL)**	124.1 ± 100.2	136.6 ± 78.5
**HDL-c (mg/dL)**	55.8 ± 13.5	43.4 ± 11.0 ^1^
**LDL-c (mg/dL)**	121.4 ± 26.9	122.4 ± 29.6
**Insulin (μIU/mL)**	13.5 ± 9.5	23.2 ± 14.1 ^1^
**HOMA-IR**	2.0 ± 2.1	5.5 ± 3.4 ^1^
**Leptin (ng/mL)**	14.8 ± 15.5	68.6 ± 35.0 ^2^
**Adiponectin (µg/mL)**	25.4 ± 16.0	9.6 ± 4.4 ^2^
**Oxidized LDL (mU/L)**	52,977 ± 13,722	73,598 ± 26,506 ^1^

The results are given as the mean ± standard deviation. BMI: body mass index. HOMA-IR: homeostasis model assessment of insulin resistance index. Significant differences between non-obese subjects and patients with morbid obesity (^1^ *p* < 0.05; ^2^ *p* < 0.001).

## Data Availability

The data presented in this study are available on request from the corresponding author. The data are not publicly available due to ethical reasons.
